# A small molecule inhibitor of tropomyosin dissociates actin binding from tropomyosin-directed regulation of actin dynamics

**DOI:** 10.1038/srep19816

**Published:** 2016-01-25

**Authors:** Teresa T. Bonello, Miro Janco, Jeff Hook, Alex Byun, Mark Appaduray, Irina Dedova, Sarah Hitchcock-DeGregori, Edna C. Hardeman, Justine R. Stehn, Till Böcking, Peter W. Gunning

**Affiliations:** 1 School of Medical Sciences, University of New South Wales, Sydney, NSW, 2052, Australia; 2 School of Medical Sciences and ARC Centre of Excellence in Advanced Molecular Imaging, University of New South Wales, Sydney, NSW, 2052, Australia; 3 Pathology & Laboratory Medicine, Robert Wood Johnson Medical School, Rutgers University, Piscataway, NJ 08854, USA

## Abstract

The tropomyosin family of proteins form end-to-end polymers along the actin filament. Tumour cells rely on specific tropomyosin-containing actin filament populations for growth and survival. To dissect out the role of tropomyosin in actin filament regulation we use the small molecule TR100 directed against the C terminus of the tropomyosin isoform Tpm3.1. TR100 nullifies the effect of Tpm3.1 on actin depolymerisation but surprisingly Tpm3.1 retains the capacity to bind F-actin in a cooperative manner. *In vivo* analysis also confirms that, in the presence of TR100, fluorescently tagged Tpm3.1 recovers normally into stress fibers. Assembling end-to-end along the actin filament is thereby not sufficient for tropomyosin to fulfil its function. Rather, regulation of F-actin stability by tropomyosin requires fidelity of information communicated at the barbed end of the actin filament. This distinction has significant implications for perturbing tropomyosin-dependent actin filament function in the context of anti-cancer drug development.

Tropomyosins (Tpms) form end-to-end polymers along actin filaments and determine the functional properties of the filament in fungi^1^, flies^2^ and mammals^3^. They belong to a highly conserved family of proteins with the greatest sequence divergence occurring at the N- and C-terminal ends due to alternative promotor use and exon splicing^4^. The N- and C-termini of adjacent Tpm molecules form an overlap complex that is required for Tpm to form cables along both sides of the helical actin filament^5^. It is not clear how the isoform-specific sequence information contained within the overlap complex contributes to differences in the way Tpms bind to and regulate actin.

Functionally distinct actin filament populations, characterised by their Tpm isoform composition, directly regulate a wide range of physiological processes in mammals^6^. In malignancy the Tpm profile is significantly altered, concomitant with dramatic rearrangements in actin cytoskeleton architecture^7^. Despite a down-regulation in high-molecular weight Tpm isoforms, actin filaments incorporating the low molecular weight isoform Tpm3.1 persist in all malignant cell types and are required for tumour cell survival in, at least, melanoma and neuroblastoma^8^,^9^. Studies implicating Tpm3.1-containing actin filaments in focal adhesion stability^10^, ERK mediated proliferation^11^ and myosin-dependent mechanical tension^12^ may speak to the specific reliance on Tpm3.1 in malignancy. How Tpm3.1 achieves these isoform-specific functions at the molecular level remains unknown.

We reported the preferential targeting of Tpm3.1-containing actin filaments by the small molecule TR100 *in vivo*^9^. We found that the actin filament disrupting effect of TR100 was shown to specifically depend on the C terminus of Tpm3.1, encoded by exon 9d, an alternatively spliced exon. Cells transfected with a Tpm3.1 chimera containing an alternative striated muscle-specific C terminus (exon 9a), demonstrated significant resistance to the effects of TR100. In this report we use TR100 as a molecular tool to dissect-out the contribution of the Tpm overlap region to actin filament regulation. We find that TR100 perturbs Tpm3.1-regulated actin filament depolymerisation from the barbed end without affecting the capacity of Tpm3.1 to regulate barbed end actin elongation or bind F-actin. The distinction made between actin disassembly and actin binding, using Tpm3.1 as proof-of-principle, may be intrinsic to all Tpm isoforms and provide an explanation for the functional significance of sequence variation in the overlap region.

## Results

**Figure 1:**
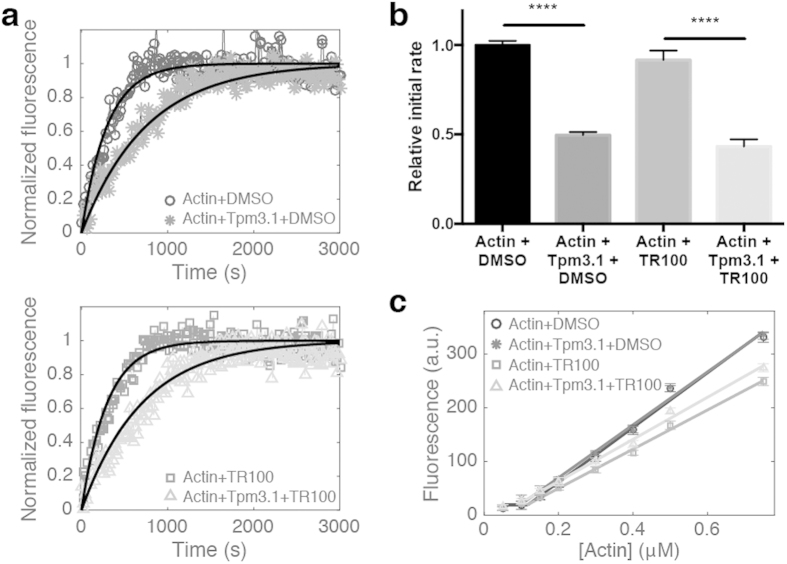
Slowed actin elongation by Tpm3.1 is not reversed in the presence of anti-Tpm compound TR100. (**a**) Representative polymerisation curves for 7 μM G-actin (10% pyrene labelled) alone or in the presence of saturating amounts (14 μM) of Tpm3.1 in 100 mM KCl, 2 mM Tris-HCl pH 8.0, 2 mM MgCl_2_, 0.2 mM EGTA, 0.2 mM ATP, 0.2 mM DTT. Tpm3.1 was pre-incubated with 50 μM TR100 or 1% (v/v) DMSO (vehicle control) prior to mixing with G-actin. Data fitted to a one phase association exponential model. (**b**) Initial rates of elongation. Rate data shown is mean ± S.D., averaged from n = 4–6 repeats. (**c**) Critical concentration of ATP-actin in the absence and presence of saturating concentrations of Tpm3.1 pre-incubated with 50 μM TR100 or 1% (v/v) DMSO (vehicle control).

Both muscle and non-muscle Tpm isoforms have been characterised for their ability to regulate actin filament kinetics^13^,^14^,^15^. It has been suggested that inhibition of actin polymerisation by Tpm may be a direct result of changing the barbed end elongation rate^14^,^15^ or the ability of Tpm to promote end-to-end annealing of actin filaments, effectively reducing the number of barbed ends primed for actin monomer addition^15^,^16^. Here we find that saturating concentrations of Tpm3.1 slow the incorporation of pyrene-labelled actin monomers into the growing filament when compared to the spontaneous polymerisation of actin alone (Fig. 1a,b). The capacity of Tpm3.1 to regulate actin polymerisation was investigated in the presence of TR100. Pre-incubation of Tpm3.1 with TR100 prior to mixing with G-actin and initiating polymerisation did not affect its ability to reduce actin polymerisation (Fig. 1a). The initial rate of actin polymerisation was approximately half the rate observed for actin alone both in the presence and in the absence of TR100 (Fig. 1b).

The critical concentration of actin assembly is the concentration of monomeric actin at steady state when the rates of polymerisation and depolymerisation are equal such that there is no net filament growth. It is determined by rates of binding and dissociation of ATP-actin at the filament ends. As observed for other Tpm isoforms^17^,^18^, the critical concentration (~0.1 μM for the barbed end) was not affected in the presence of saturating concentrations of Tpm3.1 (Fig. 1c) and TR100 did not affect critical concentration when pre-incubated with Tpm3.1 prior to binding to actin filaments (Fig. 1c).

**Figure 2:**
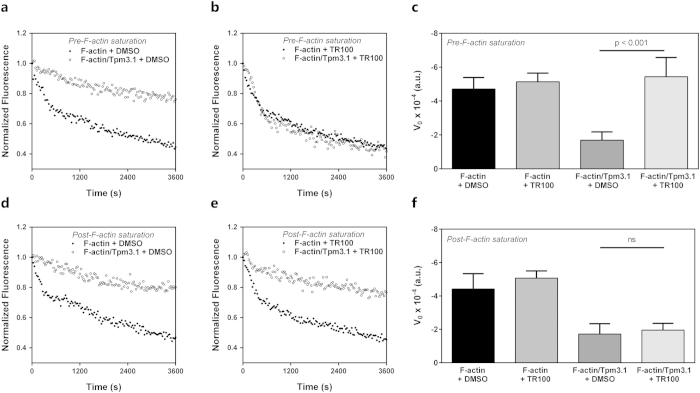
The Tpm3.1 dimer brings TR100 into the filament leading to enhanced actin depolymerisation. (**a,b,d,e**) Representative depolymerisation time course of 3 μM actin filaments (35% pyrene labelled) diluted 12-fold into F-actin buffer (100 mM NaCl, 10 mM Tris-HCl pH 7.0, 2 mM MgCl_2_, 1 mM EGTA, 0.2 mM CaCl_2_, 0.2 mM ATP, 0.5 mM DTT, 0.01% (v/v) NaN_3_) in the presence or absence of saturating amounts (5 μM) of Tpm3.1. Final concentrations of F-actin and Tpm3.1 are 0.25 μM and 0.42 μM, respectively. Tpm3.1 was pre-incubated with 1% (v/v) DMSO (**a**) or 50 μM TR100 (**b**) prior to mixing with F-actin (pre-saturation) compared to vehicle/drug added to Tpm3.1-coated F-actin (post-saturation; **d** and **e**, respectively). Depolymerisation curves are normalised to the initial fluorescence value. (**c** and **f**) Initial rates of depolymerisation, calculated from the first 600 s of depolymerisation, for F-actin alone or Tpm3.1/F-actin in the presence of vehicle or TR100. Rate data represents mean ± SEM, averaged from n = 4–6 replicates.

In addition to actin elongation kinetics Tpms have also been shown to inhibit actin filament depolymerisation^19^,^20^,^21^ and this property was recently extended to Tpm3.1^9^,^22^. We previously reported that the stabilising effect of Tpm3.1 on actin filament depolymerisation is negated by pre-incubating Tpm3.1 with TR100 prior to filament saturation^9^. We questioned whether this effect would manifest if the actin filament was initially saturated with Tpm3.1 prior to drug exposure. To address this we compared the effect of TR100 on actin filament stability pre- and post-saturation with Tpm3.1 (Fig. 2). As demonstrated previously, dilution-induced depolymerisation of F-actin, which is dominated by dissociation of ADP-actin from the barbed end of the filament, was significantly slower for filaments coated with Tpm3.1 (DMSO control, Fig. 2a,d). Pre-incubating Tpm3.1 with 50 μM TR100 prior to polymer formation largely negated the effect of Tpm3.1 on actin depolymerisation (Fig. 2b) and the initial depolymerisation rate (Fig. 2c). As a consequence, Tpm3.1/actin filaments depolymerised to a similar extent as bare actin filaments. This effect on depolymerisation however, did not manifest when F-actin filaments, fully coated with Tpm3.1 and exposed to 50 μM TR100 for 10 minutes, were diluted into F-actin buffer to initiate depolymerisation (Fig. 2d–f). This suggests that TR100 binds to Tpm free in solution and that the drug binding site is inaccessible once Tpm has polymerised on the actin filament. Furthermore, these data suggests that the functionality of the head-to-tail overlap complex makes a significant contribution to the actin-filament stabilising property of Tpm.

**Figure 3:**
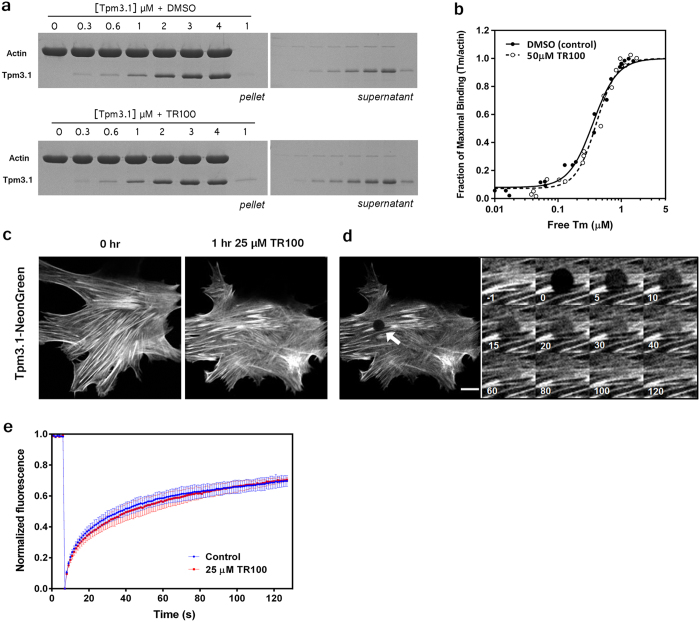
TR100 does not affect the binding capacity of Tpm3.1 for F-actin. (**a**) Increasing amounts of Tpm3.1 (0.2–5.5 μM) were pre-incubated with 1% (v/v) DMSO (vehicle control) or 50 μM TR100 prior to combining with 3 μM F-actin. The Tpm/F-actin mixture was sedimented by high-speed centrifugation at 20 °C in 150 mM NaCl, 10 mM Tris-HCl pH 7.0, 2 mM MgCl_2_ and 0.5 mM DTT. Shown are representative Coomassie-stained gels of separated pellet and supernatant fractions. (**b**) The ratio of Tpm:actin in the pellet, normalised to the ratio that occurs at Tpm saturation, plotted against the concentration of unbound Tpm in the supernatant. Data points were combined from n = 3 independent experiments. Binding constant (K_app_ × 10^6^ M^−1^) and Hill coefficient (α^H^) values (DMSO vs. TR100: K_app_ 0.36 vs. 0.40, α^H^ 2.26 ± 0.22 vs. 2.71 ± 0.28). (**c**) Loss of Tpm3.1-mNeonGreen-containing stress fibers in mouse embryonic fibroblasts (MEFs) treated with 25 μM TR100 for 1 h. (**d**) Time-lapse images of fluorescence recovery (initial bleach site indicated by white arrow) and (**e**) respective recovery curves of Tpm3.1-mNeonGreen-containing stress fibers in MEFs treated with 25 μM TR100 for 1 h. Data generated from n = 3 experiments (5–15 cells per experiment). Scale bar, 10 μM.

It is possible that the impact of TR100 on Tpm-regulated actin filament kinetics reflects a change in the binding capacity of Tpm3.1 for actin. To investigate this the affinity of Tpm3.1 for F-actin was measured by co-sedimentation. Increasing concentrations of Tpm3.1 were mixed with a constant amount of F-actin and pelleted by high speed centrifugation to separate out the actin-bound (pellet) and unbound (supernatant) fractions of Tpm3.1 (Fig. 3a). As expected, Tpm3.1 bound to skeletal F-actin with positive cooperativity as indicated by the sigmoidal shape of the binding-curve (Fig. 3b). Of note, we predetermined that Tpm3.1 binds to non-muscle beta F-actin with a similar binding affinity and cooperativity (alpha-actin k_app_ 0.46 ± 0.11, α^H^ 1.8 ± 0.6 vs. beta-actin k_app_ 0.48 ± 0.80, α^H^ 2.5 ± 1.0, data not shown). Surprisingly, at an identical concentration of TR100 used to elicit effects on Tpm3.1-regulated actin depolymerisation (50 μM), we did not observe any effect on the affinity of Tpm3.1 for F-actin or the cooperativity of binding (Fig. 3a,b). This indicates, that despite changes to Tpm3.1-regulated actin filament kinetics in the presence of TR100, Tpm3.1 does not lose the capacity to bind at the actin interface in a cell-free assay.

To corroborate these findings *in vivo,* FRAP analysis was employed to measure the recovery kinetics of tagged Tpm3.1 following bleaching. Mouse embryonic fibroblasts (MEFs) transfected with Tpm3.1-mNeonGreen were treated with 25 μM TR100 for 1 hour prior to FRAP analysis. At this concentration and treatment time an obvious change in cell morphology was observed (Fig. 3c). Specifically cells became less spread and there was a reduction in the appearance of large actin bundles with TR100 treatment. Time-lapse recordings of Tpm3.1-containing stress fibers following photobleaching show that TR100 does not affect the exchange of Tpm3.1 into filaments (Fig. 3d) or the recovery kinetics (Fig. 3e). Together, the co-sedimentation and FRAP data indicate that Tpm3.1 binds to actin equally well in the presence and absence of TR100.

## Discussion

We propose that TR100 acts to compromise the integrity of Tpm cables rather than prevent overlap complex formation. Our data suggests that TR100 is incorporated into the growing actin-Tpm co-polymer given that its effects cannot be observed on pre-formed Tpm3.1/actin filaments. Certainly, Tpm3.1 can form a continuous polymer with actin in the presence of TR100 which must involve both Tpm3.1-actin binding and Tpm3.1 head-to-tail cooperative binding^23^. These results therefore dissociate the ability of Tpm3.1 to bind along an actin filament from its ability to regulate actin filament stability.

The C terminus of Tpm is helical and a coiled coil but contains a hinge near the end to enable the helical ends to splay apart and form the overlap complex with the coiled coil N terminus. The C terminus must be flexible in order to interact with the N terminus^24^. Upon formation of the overlap complex both ends are stabilised, though the overlap complex remains dynamic^25^,^26^. Therefore, we propose a mechanism of action in which TR100 binds to the uncomplexed C terminus of Tpm3.1 in a conformation permissible for N-terminal binding. It is a formal possibility that the presence of C-terminal bound TR100 introduces steric hindrance in the overlap complex leading to reduced flexibility in this region. In both striated and smooth muscle isoforms the overlap domain is characterised by a degree of flexibility^25^,^27^,^28^,^29^ which is likely a governing factor in how information is communicated along the Tpm polymer as well as between the Tpm polymer and the actin filament. Finally, given that the binding capacity of Tpm3.1 for actin is unaffected by TR100, exactly how actin dynamics is altered remains a subject of intense interest. One possibility is the existence of different conformational states of actin induced by Tpm binding^23^. Due to the highly cooperative nature of the actin polymerisation/depolymerisation process, small conformational changes to the actin filament would likely result in dramatic changes to kinetic assembly and disassembly.

Unlike striated muscle Tpm which requires N-terminal acetylation to associate end-to-end and for cooperative binding to filamentous actin^30^, bacterially expressed non-muscle Tpms are capable of self-association and actin binding in the absence of acetylation^31^,^32^,^33^. Given that N-terminal acetylation has been shown to stabilise the alpha-helical conformation of an N-terminal striated muscle peptide, albeit with little effect on the overall stability of full length Tpm^34^, it may still be important to investigate whether this conformational change extends to non-muscle Tpm isoforms and the potential implications for cooperative binding to actin. It should be noted however, that in the fission yeast model, both acetylated and non-acetylated subpopulations of a single Tpm isoform (Cdc8) exist and are associated with distinct actomyosin pools in the cell^35^. It is therefore possible that acetylation status serves a similar purpose amongst mammalian Tpm isoforms, making it important to compare the effects of anti-Tpm compounds on both modified and non-modified forms of Tpm.

Concomitant with disrupted actin filament integrity *in vivo*, TR100 demonstrates strong cytotoxicity against neuroblastoma and melanoma cell lines grown in 2D and 3D culture^9^. Interestingly, the dose-response curves for TR100 are characterised by a steep slope, indicating a narrow concentration range over which TR100 acts to reduce cell viability. This threshold response may correspond to loading a sufficient amount of TR100 into the filament, which correlates with our current finding that TR100 is gradually incorporated into the polymerising actin-Tpm3.1 filament. This would suggest that the threshold is based on two states of the filament; functional versus non-functional, rather than TR100 having a graded effect on filament function.

Tpm3.1 localises strongly to stress fibers and both nascent and mature focal adhesions^36^,^37^, however a clear consensus on the preferential association of Tpm3.1 for beta versus gamma-actin does not exist in an absolute sense. The association of Tpm3.1 with either actin isoform appears to be cell type specific^38^,^39^. Nevertheless, the demonstration that beta and gamma actin have intrinsic functional differences in their ability to activate different myosin II motors opens the possibility that Tpm3.1-containing filaments composed of different actins may respond differently to TR100 in different cellular contexts^40^.

## Methods

### Reagents

TR100 was designed by T.A. Hill and A. McCluskey as described previously^9^. TR100 was synthesised by SynMedChem and prepared as a 50 mmol/L stock in DMSO.

### Protein purification

Rabbit skeletal muscle actin was sourced commercially (Cytoskeleton Inc.), with the exception of actin used in the spontaneous actin polymerisation and critical concentration assay which was prepared and labelled with pyrene as described previously^41^.

The human homologue of Tpm3.1 was expressed in *E. coli* BL21 (DE3) cells and purified according to Fanning *et al*., 1994^32^ with modifications. Briefly, recombinant Tpm3.1 was purified by ammonium sulphate precipitation and anion exchange chromatography.

### Actin polymerisation and depolymerisation assay

The rates of actin polymerisation and depolymerisation were determined from the change in pyrene-actin fluorescence (excitation 365 nm, emission 407 nm) measured using a Spectra Max M3 plate reader (Molecular Devices; polymerisation) and EnSpire multimode plate reader (Perkin Elmer; depolymerisation). For spontaneous actin polymerisation, 7 μM Mg-G-actin (10% pyrenylactin) and 14 μM Tpm3.1, prepared in 2 mM Tris-HCl pH 8.0, 0.2 mM ATP, 0.1 mM CaCl_2_, and 0.2 mM DTT, were assembled in a 96 well black plate. Polymerisation was initiated by the addition of 100 mM KCl and 2 mM MgCl_2_ (final concentration). Fluorescence measurements were taken every 30 s for 1 h. Fluorescence values were corrected for baseline fluorescence by subtracting G-actin and buffer values alone. Normalised data were fit to a one-phase association exponential model (MATLAB) and the K values taken as the initial polymerisation rate.

For depolymerisation assays 3 μM F-actin (35% pyrenylactin) and 5 μM Tpm3.1 were pre-incubated for 30 min. For experiments including TR100, Tpm3.1 was incubated with 50 μM compound for 10 min prior to mixing with F-actin (pre-saturation) or F-actin/Tpm3.1 filaments were incubated with compound for 10 min (post-saturation). The F-actin/Tpm3.1 samples were transferred to a 96 well black plate and depolymerisation was initiated by diluting filaments 12 fold with F-actin buffer (100 mM NaCl, 10 mM Tris-HCl pH 7.0, 2 mM MgCl_2_, 1 mM EGTA, 0.2 mM ATP, 0.5 mM DTT). Fluorescence was recorded every 30 s over 1 h. Fluorescence values were corrected for background fluorescence from buffer and TR100 alone and normalised to the initial fluorescence value at time 0 s. Initial rates of depolymerisation were determined from the first 600 s of fluorescence, fitted to a linear regression model.

Critical concentration of actin was measured according to method of Lal and Korn^17^. Briefly, fluorescence of actin filaments (30% pyrenyl-labelled actin) in steady state were measured using an EnSpire multimode plate reader (Perkin Elmer). The excitation and emission wavelengths were 365 nm and 407 nm, respectively. Firstly, a 3 μM F-actin stock was prepared by polymerisation of Mg-G-actin for 1 h at 25 °C. Polymerisation was initiated by addition of 100 mM NaCl and 2 mm MgCl_2_ (final concentration). Samples containing Tpm3.1 were mixed with actin in excess concentration of 5 μM. Additionally, Tpm3.1 was incubated with 50 μM TR100 for 30 min prior to initiating polymerisation. Polymerised actin was then diluted to a range of concentrations between 1.5 – 0.05 μM and incubated for 18 h at 25 °C to reach steady state.

### Actin/Tpm co-sedimentation assay

The affinity of Tpm for F-actin was measured by co-sedimentation according to Heald and Hitchcock^42^, with modifications. Prior to co-sedimentation, Tpm3.1 was pre-incubated with 50 μM TR100 compound or 1% (v/v) DMSO vehicle control for 10 min. Increasing concentrations of Tpm (0.2–5.5 μM) were mixed with 3 μM rabbit skeletal F-actin in buffer containing 150 mM NaCl, 10 mM Tris-HCl pH 7.0, 2 mM MgCl_2_ and 0.5 mM DTT. Samples were incubated for 20 min at room temperature before pelleting at 50,000 rpm for 30 min at 20 °C. Supernatant and pellet were resolved on a 12.5% polyacrylamide gel and proteins visualised with Coomassie Blue. Gels were scanned using a LAS4000 imager and densitometry was performed in Image J (Image J software, NIH). The ratio of Tpm to actin in the pellet was normalised to 1.0 by dividing the Tpm to actin ratio obtained from densitometry by the observed maximal ratio at saturation. The concentration of free Tpm in the supernatant was calculated from a standard curve of Tpm run in parallel with the samples. The Tpm to actin ratio was plotted against the concentration of free Tpm. The binding constant (K_app_) of Tpm for actin and the Hill coefficient (α^H^) were determined by fitting the experimental data to a non-linear regression model (4 parameter, variable slope) in GraphPad Prism 5.0.

### Fluorescence recovery after photobleaching

To create the Tpm3.1-mNeonGreen construct, human Tpm3.1 cDNA (NCBI mRNA accession# NM_153649) followed by the sequence encoding mNeonGreen at the C-terminus (a gift from Jiwu Wang), separated by a linker motif (GGGGSGGGGS), were subcloned into pcDNA3.1 containing a CMV promotor (GeneArt, Invitrogen). Primary MEFs were maintained in Dulbecco’s modified Eagle medium (DMEM) supplemented with 10% foetal bovine serum (FBS) at 37 °C, 5% CO_2_. Transfection with Tpm3.1-mNeonGreen construct was performed using Lipofectamine 3000 reagent (Life Technologies) according to manufacturer’s instructions. Tpm3.1-mNeonGreen expressing MEFs were used for imaging on days 2 and 3 post-transfection. Live cell imaging of transfected MEFs was performed on a Nikon A1 inverted scanning confocal microscope equipped with NIS Elements software, incubation chamber (37 °C) and CO_2_ control. Photobleaching was achieved with a single 120.38 ms pulse using a 488-nm laser, focused using a Nikon Plan Apochromat λ 60×/1.4 (oil) objective. For FRAP analyses, a circular region of interest (5 μM diameter), containing mostly stress fibers, was selected for photobleaching per cell. Pre-bleached scans (3–5 per cell) were taken to determine initial fluorescence intensity. Following a single bleach pulse images were acquired every 1 s for 2 min. To obtain control FRAP curves, select zones containing stress fibers were first photobleached prior to the addition of TR100. Cells were then incubated with 25 μm TR100 for 1 h and the same cell was photobleached at an alternate target zone containing stress fibers. FRAP curves generated were normalised to minimum and maximum fluorescence values. Data from normalised FRAP curves were processed to obtain standard error measurement values for each respective time point.

## Additional Information

**How to cite this article**: Bonello, T. T. *et al*. A small molecule inhibitor of tropomyosin dissociates actin binding from tropomyosin-directed regulation of actin dynamics. *Sci. Rep.* **6**, 19816; doi: 10.1038/srep19816 (2016).
